# Reviews

**Published:** 1882-12

**Authors:** 


### Reviews.

**The Diseases of the Liver, with and without Jaundice, with Special Application of Physiological Chemistry to their Diagnosis and Treatment.** By GEORGE HARLEY, M. D., F. R. S., Fellow College Physicians and Professor in University College, London. Illustrated by colored plates and wood engravings. Philadelphia: P. Blakiston, Son & Co., 1012 Walnut St. 1883.

This work is the outcome of a wide and varied study of the liver, both in health and in disease. It aims to base the diagnosis and treatment of hepatic diseases upon rational and scientific principles, by carrying physiological chemistry into the domain of medical science. The author emphasizes the value and importance of a knowledge of healthy function, and strives to trace morbid phenomena through the avenues afforded by the chemical analysis of the excretions. He believes that by this method we are entering upon an "important line of medical inquiry, which, sooner or later, will be followed by valuable practical results." The older authors were unsatisfactory in their pathology and empirical in their treatment of hepatic diseases. They quite uniformly started off in their attempted elucidation of symptoms by enlarging upon the obscurity and vagueness of all diseases of the liver. The author of this work analyzes every disease, traceable to an altered condition of this important organ, by utilizing all the means which chemistry and physiology furnish. As a consequence, the work is the clearest and most satisfactory of any we have examined.

Inasmuch as the work is not intended for the medical student, but rather for medical men of experience and education, its author very wisely has added a chapter in which he summarizes the differential diagnosis of diseases of the liver, and thus condenses in a brief space the essential symptoms peculiar to each morbid condition. We regard this chapter to the busy practitioner a valuable addition, and it increases the real worth to those whose time is limited for study.

We need not refer further to other features of the work, which strike us favorably as we peruse its pages. In those sections of our country in which hepatic diseases prevail through climatic influences and causes, the profession can not afford to be without such an aid as this presents for their study and investigation. It is the most thoroughly scientific work ever offered to the profession in the diagnosis and treatment of diseases of the liver.

---

**Pharmacopœia of the United States of America**. Sixth Decennial Revision. By authority of the National Convention for Revising the Pharmacopœia, held at Washington, D. C., 1880. New York: William Wood & Co. 1882.

On opening this valuable work, we are favored with a brief historical introduction of the measures adopted in the past for the formation of a National Pharmacopœia. In this important movement, inaugurated in January, 1817, by Dr. Lyman Spalding, who submitted to the Medical Society of the County of New York a project looking to this end, we find engaged, successively, such able Pharmacologists as Dr. Samuel L. Mitchell, who became President of the General Convention for the formation of a National Pharmacopœia in 1820; Dr. Lewis Condict, of New Jersey, who was President in 1830; Dr. Geo. B. Wood, President in 1850; Dr. Joseph Carson, in 1870. Following this historical introduction is the Abstract of the Proceedings of the National Convention of 1880 for Revising the Pharmacopœia, of which Dr. Robert Amory, of Massachusetts, was President. This convention, after laying out its work, made provision for calling a convention in 1890.

The work here presented is the most complete of any of its predecessors in the precision of its details, as well as in the extent of the subjects treated. It furnishes a complete Pharmacopœia, with the addition of all the new remedies presented during the last decade, with list of reagents or articles used in testing, table of thermometric equivalents, table of percentage and specific gravity, table of the solubility of chemicals in water and alcohol, saturation table, list of articles added to and dismissed from the Pharmacopœia, etc., etc. It will be seen that the work is a valuable addition to the literature of this important department of medicine. It is presented on good paper and excellent type, and reflects great credit upon the enterprising publishers.

---

**A Guide to Therapeutics and Materia Medica.** By ROBT. FARQUHARSON, M. D., Edin., F. R. C. P., Lond., late lecturer on Materia Medica at St. Mary's Hospital Medical School. Third American edition, revised by the author; enlarged and adapted to the U. S. Pharmacopœia by FRANK WOODBURY, M. D., Physician to the German, Hospital Philadelphia. H. C. Lea's Son & Co.

As "good wine needs no bush," "Farquharson's Therapeutics" needs no commendation. We are glad, however, to see a third edition issued under the editorship of Dr. Woodbury. In the previous edition he greatly added to the value of the work, to the American reader, by adapting it to our Pharmacopœia. In the present edition, in deference to the demands of of scientific progress, the metric system has been introduced in addition to the old form of writing prescriptions. The tests of the permanent poisons have also been inserted in the text, and a ready reference table of poisons at the end of the book.

---

**The Principles and Practice of Surgery.** By JOHN ASHHURST, JR., M. D., Professor of Clinical Surgery in the University of Pennsylvania, Senior Surgeon to the Children's Hospital, Consulting Surgeon to the Woman's Hospital, etc., etc. Third edition. Enlarged and thoroughly revised, with five hundred and fifty-five illustrations. Philadelphia: H. C. Lea's Son & Co. 1882.

The opinion, expressed in the first appearance of this great work, that it would take rank among the best works in surgery in the world, has been fully verified, and we are glad to see a third edition, the improvements in which are modestly referred to in the following extract from the preface: "The object of this work is, as its title indicates, to furnish, in as concise a manner as may be compatible with clearness, a condensed but comprehensive description of the modes of practice now generally employed in the treatment of surgical affections, with a plain exposition of the principles upon which those modes of practice are based. In revising his work for a third edition, the author has spared no pains to render it worthy of a continuance of the favor with which it has heretofore been received, by incorporating in it an account of the more important recent observations in surgical science, and of such novelties in surgical practice as have seemed to him to be really improvements; and by making such changes as have been suggested to him by enlarged personal experience as a clinical teacher and hospital surgeon." To the general practitioner, who has not the time to give attention to the more minute and extended works, and to the medical student, we would most cordially commend it. Like all of Lea's books, it is an illustration of the perfection of book-making. The new style of half Russia binding is particularly attractive. The price of the book is very low; cloth, $6.00; leather, $7.00; half Russia, $7.50.

---

**The Medical Record Visiting List or Physician's Diary for 1883.** New York: William Wood & Co.

This visiting list is issued in the usual style and will be found a valuable aid in the daily record it furnishes for the physician.

---

**Handbook of Homœopathic Practice.** By George M. Ockford, M. D. Chicago: Duncan Brothers. 1882.

In the preface of this work we learn that "one main object of the compilation of this handbook is to present, in a concise form, practical descriptions of the principal diseases and their treatment. It is a series of notes culled from the writings of foreign and American authorities, and is issued with the hope that it may prove useful to the student of homœopathy as well as to the busy practitioner to whom time is too valuable to ponder over the larger works of his library." The book is apparently what the preface leads us to expect. The symptoms of disease are, on the whole, concisely and correctly given. As to the treatment, our knowledge is limited and eclipsed by our skepticism. The chapter on mental disorders is particularly startling in its claims for the potency and applicability of drugs.

